# Impact of individual level uncertainty of lung cancer polygenic risk score (PRS) on risk stratification

**DOI:** 10.1186/s13073-024-01298-4

**Published:** 2024-02-05

**Authors:** Xinan Wang, Ziwei Zhang, Yi Ding, Tony Chen, Lorelei Mucci, Demetrios Albanes, Maria Teresa Landi, Neil E. Caporaso, Stephen Lam, Adonina Tardon, Chu Chen, Stig E. Bojesen, Mattias Johansson, Angela Risch, Heike Bickeböller, H-Erich Wichmann, Gadi Rennert, Susanne Arnold, Paul Brennan, James D. McKay, John K. Field, Sanjay S. Shete, Loic Le Marchand, Geoffrey Liu, Angeline S. Andrew, Lambertus A. Kiemeney, Shan Zienolddiny-Narui, Annelie Behndig, Mikael Johansson, Angie Cox, Philip Lazarus, Matthew B. Schabath, Melinda C. Aldrich, Rayjean J. Hung, Christopher I. Amos, Xihong Lin, David C. Christiani

**Affiliations:** 1 Department of Environmental Health, Harvard T.H. Chan School of Public Health, Harvard University, 667 Huntington Ave, Boston, MA 02115 USA; 2 Department of Medical Oncology, Dana-Farber Cancer Institute, Boston, MA USA; 3 Bioinformatics Interdepartmental Program, University of California, Los Angeles, USA; 4 Department of Biostatistics, Harvard T.H. Chan School of Public Health, Harvard University, Boston, MA USA; 5 Department of Epidemiology, Harvard T.H. Chan School of Public Health, Harvard University, Boston, MA USA; 6 Division of Cancer Epidemiology and Genetics, National Cancer Institute, National Institutes of Health, Bethesda, MD USA; 7 Department of Medicine, British Columbia Cancer Agency, University of British Columbia, Vancouver, Canada; 8 Faculty of Medicine, University of Oviedo and CIBERESP, Oviedo, Spain; 9 Department of Epidemiology, University of Washington School of Public Health, Public Health Sciences Division, Fred Hutchinson Cancer Research Center, Seattle, WA USA; 10 Department of Clinical Biochemistry, Herlev and Gentofte Hospital, Copenhagen University Hospital, Copenhagen, Denmark; 11 Genomic Epidemiology Branch, International Agency for Research on Cancer (IARC/WHO), Lyon, France; 12 Department of Biosciences and Medical Biology, Allergy-Cancer-BioNano Research Centre, University of Salzburg, and Cancer Cluster Salzburg, Salzburg, Austria; 13 Department of Genetic Epidemiology, University Medical Center, Georg August University Göttingen, Göttingen, Germany; 14 Institute of Medical Informatics, Biometry and Epidemiology, Ludwig Maximilians University, Munich, Germany; 15 Clalit National Cancer Control Center, Carmel Medical Center and Technion Faculty of Medicine, Carmel, Haifa, Israel; 16 Markey Cancer Center, University of Kentucky, Lexington, KY USA; 17 Department of Molecular and Clinical Cancer Medicine, Institute of Translational Medicine, University of Liverpool, Liverpool, UK; 18 Department of Biostatistics, The University of Texas MD Anderson Cancer Center, Houston, TX USA; 19 Epidemiology Program, University of Hawaii Cancer Center, Honolulu, HI USA; 20 Princess Margaret Cancer Centre, Dalla Lana School of Public Health, University of Toronto, Toronto, Canada; 21 Department of Epidemiology, Department of Community and Family Medicine, Dartmouth Geisel School of Medicine, Hanover, NH USA; 22 Department for Health Evidence, Department of Urology, Radboud University Medical Center, Nijmegen, The Netherlands; 23 National Institute of Occupational Health, Oslo, Norway; 24 Department of Public Health and Clinical Medicine, Umeå University, Umeå, Sweden; 25 Department of Radiation Sciences, Umeå University, Umeå, Sweden; 26 Department of Oncology and Metabolism, The Medical School, University of Sheffield, Sheffield, UK; 27 Department of Pharmaceutical Sciences, College of Pharmacy, Washington State University, Spokane, WA USA; 28 Department of Cancer Epidemiology, H. Lee Moffitt Cancer Center and Research Institute, Tampa, Florida, USA; 29 Department of Medicine, Department of Biomedical Informatics and Department of Thoracic Surgery, Vanderbilt University Medical Center, Nashville, TN USA; 30 Lunenfeld-Tanenbaum Research Institute, Sinai Health System, Dalla Lana School of Public Health, University of Toronto, Toronto, Ontario Canada; 31 Institute for Clinical and Translational Research, Department of Medicine, Dan L Duncan Comprehensive Cancer Center, Baylor College of Medicine, Houston, TX USA

**Keywords:** Non-small cell lung cancer (NSCLC), Polygenic risk score (PRSs), Cancer control, Population science, Genetic epidemiology

## Abstract

**Background:**

Although polygenic risk score (PRS) has emerged as a promising tool for predicting cancer risk from genome-wide association studies (GWAS), the individual-level accuracy of lung cancer PRS and the extent to which its impact on subsequent clinical applications remains largely unexplored.

**Methods:**

Lung cancer PRSs and confidence/credible interval (CI) were constructed using two statistical approaches for each individual: (1) the weighted sum of 16 GWAS-derived significant SNP loci and the CI through the bootstrapping method (PRS-16-CV) and (2) LDpred2 and the CI through posteriors sampling (PRS-Bayes), among 17,166 lung cancer cases and 12,894 controls with European ancestry from the International Lung Cancer Consortium. Individuals were classified into different genetic risk subgroups based on the relationship between their own PRS mean/PRS CI and the population level threshold.

**Results:**

Considerable variances in PRS point estimates at the individual level were observed for both methods, with an average standard deviation (s.d.) of 0.12 for PRS-16-CV and a much larger s.d. of 0.88 for PRS-Bayes. Using PRS-16-CV, only 25.0% of individuals with PRS point estimates in the lowest decile of PRS and 16.8% in the highest decile have their entire 95% CI fully contained in the lowest and highest decile, respectively, while PRS-Bayes was unable to find any eligible individuals. Only 19% of the individuals were concordantly identified as having high genetic risk (> 90th percentile) using the two PRS estimators. An increased relative risk of lung cancer comparing the highest PRS percentile to the lowest was observed when taking the CI into account (OR = 2.73, 95% CI: 2.12–3.50, *P*-value = 4.13 × 10^−15^) compared to using PRS-16-CV mean (OR = 2.23, 95% CI: 1.99–2.49, *P*-value = 5.70 × 10^−46^). Improved risk prediction performance with higher AUC was consistently observed in individuals identified by PRS-16-CV CI, and the best performance was achieved by incorporating age, gender, and detailed smoking pack-years (AUC: 0.73, 95% CI = 0.72–0.74).

**Conclusions:**

Lung cancer PRS estimates using different methods have modest correlations at the individual level, highlighting the importance of considering individual-level uncertainty when evaluating the practical utility of PRS.

**Supplementary Information:**

The online version contains supplementary material available at 10.1186/s13073-024-01298-4.

## Background

Lung cancer is a multifactorial disease with high incidence and mortality [1, 2]. Environmental exposures including tobacco smoking [3–6], occupational exposures [7, 8], and air pollution [9–11], as well as heritable genetics, contribute to lung cancer risk [12]. Unfortunately, a majority of lung cancer cases are diagnosed at a late disease stage with a poor 5-year survival rate of less than 5%. There is, therefore, an urgent and unmet need to detect lung cancer early when prevention or earlier intervention is possible [1]. Polygenic risk scores (PRSs) have emerged as a promising tool for predicting cancer risk from genome-wide association studies (GWAS) [13–16]. Lung cancer PRSs and their potential clinical utility have been explored in European and Chinese populations [2, 17–20]. Lung cancer PRSs may provide additional information for personalized risk stratification and prediction beyond non-genetic risk factors [17–20].

As PRS prediction moves towards clinical implementation in personalized medicine, accurate and unbiased PRS predictions for any single individual are needed. Unfortunately, to date, the predictive accuracy of PRS has been mostly evaluated at the population level using cohort-level metrics of prediction *R*^2^ and area under the curve (AUC), with its precision at a single individual level remaining largely unexplored [21, 22]. Moreover, whether individual-level PRS instability would influence the subsequent clinical utilization of PRS-based risk stratification and prediction is also of great interest to discover [21–25]. In recent studies, the individual level uncertainty in PRS estimation on various traits including height and body mass index, and diseases including breast cancer, hypertension, and dementia have been assessed in the British population using UK Biobank data [21, 22]. Another study showed that post-traumatic stress disorder and type 2 diabetes PRSs estimated among populations from different ancestries have very modest correlations at the individual level [24]. To our knowledge, there has been no study investigating the stability of lung cancer PRS for individuals and its potential impact on subsequent prediction for downstream clinical applications.

Using data from the International Lung Cancer Consortium (ILCCO) [16], we estimated lung cancer PRSs and constructed corresponding confidence/credible interval (CI) for each individual using two statistical approaches: (1) the weighted sum of 16 GWAS-derived significant single nucleotide polymorphisms (SNP) loci that have been validated in European descent population and the CI through bootstrapping method (PRS-16-CV) and (2) LDpred2 and the CI through posteriors sampling (PRS-Bayes) [21]. We further evaluated the impact of individual-level PRS uncertainty on PRS-based ranking, risk stratification, and prediction in conjunction with non-genetic risk factors of age, gender, and smoking history. Our research shows that the uncertainty of lung cancer PRS at the individual level greatly impacts the subsequent performance of individual risk stratification and prediction, highlighting the importance of cautious clinical interpretation and implementation in precision medicine.

## Methods

### Study population

**Fig. 1:**
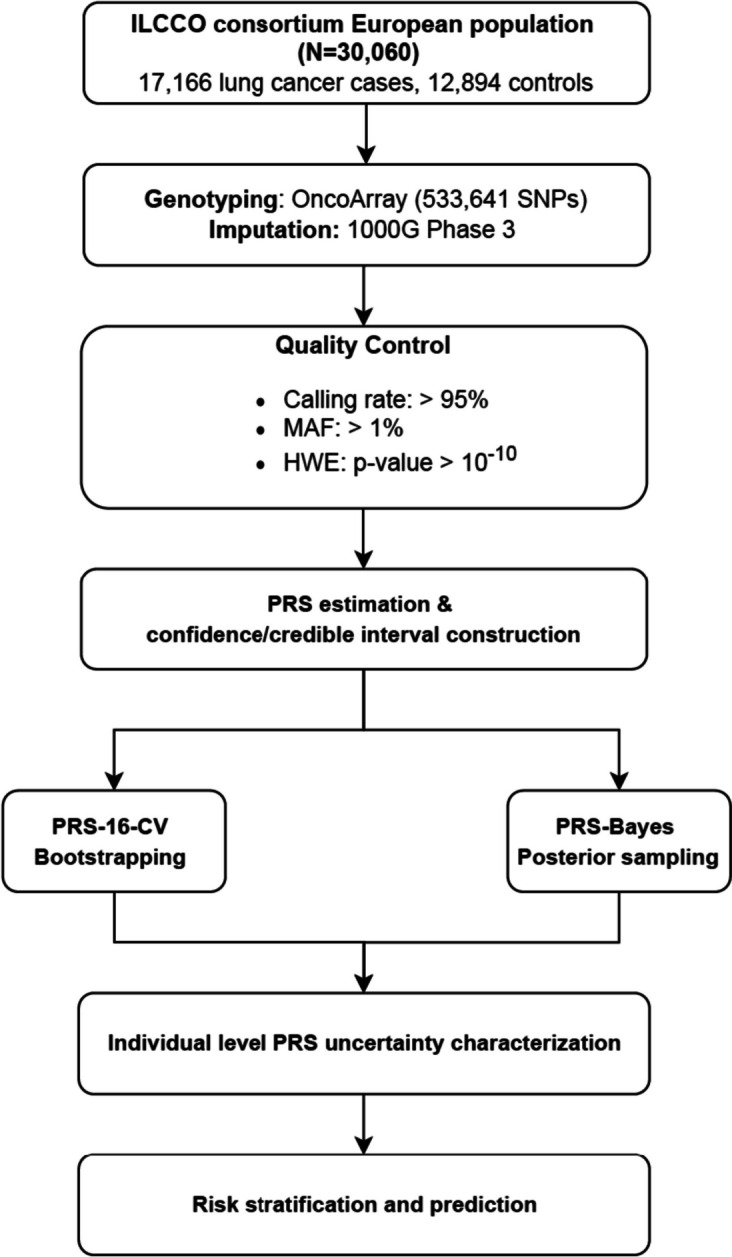
Overview of the study. The study was conducted in 17,166 lung cancer cases and 12,894 controls with European ancestry from the International Lung Cancer Consortium (ILCCO). The lung cancer PRSs and corresponding confidence/credible interval were constructed using two statistical approaches for each individual—(1) the weighted sum of 16 GWAS-derived significant SNP loci that have been validated in European descent population and the confidence interval through the bootstrapping method (PRS-16-CV) and (2) LDpred2 and the credible interval through posteriors sampling (PRS-Bayes). The individual-level PRS uncertainty was characterized and the impact on subsequent risk stratification and prediction were evaluated

We conducted our study in 30,060 participants of European ancestry (17,166 lung cancer cases and 12,894 controls) from 25 lung cancer OncoArray studies of ILCCO including both population-based and hospital-based case-control studies. The basic characteristics of these studies are summarized in Table S1 (Fig. 1, Additional file 1: Table S1). The eligibility criteria of studies to be included in ILCCO were that they had a study protocol for subject recruitment and a structured questionnaire for baseline lifestyle information. A detailed description of each study was described in the consortium flagship paper [16, 26]. Baseline demographics of age, gender, self-reported race/ethnicity, smoking history, and lung cancer histology information were collected and adjusted for in multivariable logistic regression analyses.

### Genotyping, imputation, and quality control

Genotyping was performed at the Center for Inherited Disease Research using the OncoArray platform with 533,631 SNPs from Illumina and imputation was conducted based on 1000G phase 3 reference panel [27]. Standard QC procedures were applied to keep variants with genotype calling rate > 95%, minor allele frequency > 1%, and deviation from Hardy-Weinberg equilibrium with *P*-value > 10^−10^ in controls. A total of 5,097,871 variants remained in our statistical analyses. To adjust for subtle population stratification, we performed a principal component analysis using PLINK v1.9 [28] and adopted the top 10 principal components (PCs) following reference [29].

### PRS estimation and confidence/credible interval (CI) construction

*PRS-16-CV* was calculated as the weighted sum of 16 GWAS-derived common SNP loci that have been validated in European descent populations [18]. Effect sizes were estimated through five-fold cross-validation after adjusting for age, gender, smoking status, and 10 PCs. Confidence intervals (CI) for PRS-16-CV were constructed using 1000 bootstrap samples within each fold. Therefore, each individual would have their own individual-level PRS distribution characterized by the PRS-16-CV bootstrapped mean and CI for downstream risk stratification and prediction. Cross-validation and bootstrapping were implemented using R v4.1.0. In addition, we calculated the *PRS-16* based on the same 16 SNP loci with effect sizes directly extracted from the GWAS catalog and previous literature (Additional file 1: Table S2-3), and no confidence interval was provided for the PRS-16 [16, 18, 30–33].

On the other hand, *PRS-Bayes* was estimated using a Bayesian approach leveraging the LDpred2 framework, and PRS-Bayes credible interval was constructed via posterior distribution sampling using the method proposed by Ding et al. [21, 34]. More specifically, we utilized the same training data in *PRS-16-CV* to estimate a posterior distribution of effect sizes, given the observed genotype and lung cancer status. For each variant, a sample of effect size estimates was drawn from the posterior distribution of the causal effect size using Markov chain Monte Carlo. A credible interval of the PRS-Bayes estimator was constructed by aggregating the number of effect allele copies weighted by each of the drawn estimates.

### Characterization of PRS uncertainty at the individual level

We characterized the individual level uncertainty in PRS estimation with standard deviation (s.d.) and a pre-specified confidence/credible level of CI. With a pre-specified confidence/credible level *p*, we derived an individual *p*-level CI of PRS by obtaining the empirical (1-*p*)/2 and (1+*p*)/2 quantiles from the individual level PRS distribution.

### Impact of individual-level PRS uncertainty on PRS-based risk stratification

**Fig. 2:**
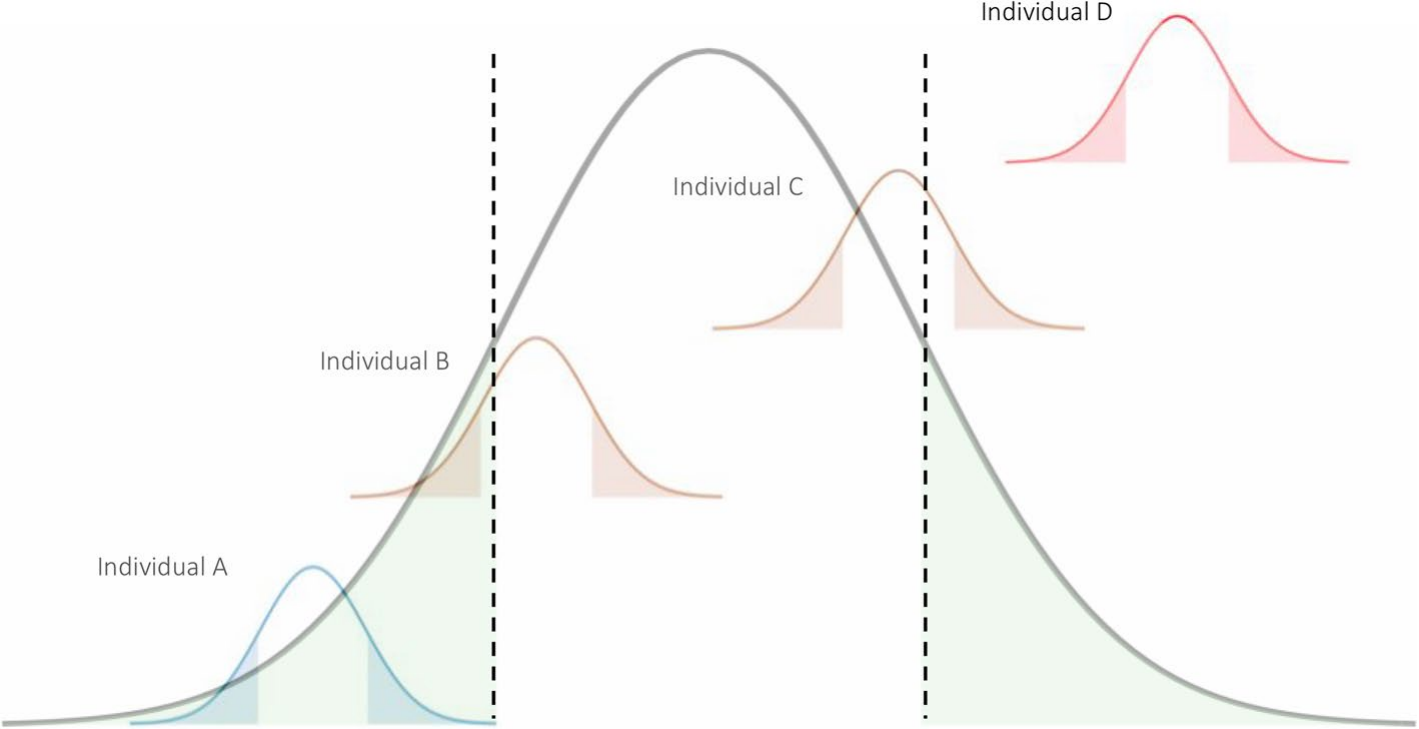
Risk stratification based on PRS confidence/credible interval (CI). The large distribution illustrates the PRS distribution at the population level, and the four small ones refer to individual PRS distributions for participants with different PRS-based risks of lung cancer. The dashed horizontal lines indicate the population level thresholds for risk stratification. Individuals with their PRS CI above a pre-specified population-level threshold *t* at the upper tail (e.g., *t* = 90th percentile) were classified as certainly high genetic risk, and similarly for individuals with PRS CI below the population level threshold *t* at the lower tail (e.g., *t* = 10th percentile) as certainly low genetic risk. Individuals whose CI covered the population level threshold were considered uncertain

Having obtained PRSs and corresponding CI, we stratified individuals into different PRS risk subgroups based on the relationship between their own PRS CI and the population level threshold (Fig. 2). Individuals with their PRS CI above a pre-specified population-level threshold *t* at the upper tail (e.g., *t* = 90th percentile) were classified as certainly high genetic risk and similarly for individuals with PRS CI below the population level threshold *t* at the lower tail (e.g., *t* = 10th percentile) as certainly low genetic risk. Individuals whose CI covered the population level threshold were considered uncertain. As a comparison, we classified individuals based on their estimated PRS mean and population threshold without taking individual-level certainty into account.

To evaluate the degree of consistency for PRS-based risk stratification, we counted the number of overlapped individuals that are concordantly identified as the same PRS-based risk by different PRS estimators. Sensitivity analyses were conducted by changing the confidence/credible level *p* and risk threshold *t* at the population level. In addition to the two PRS approaches, we also assessed the concordance of PRS-based risk stratification among 22 lung cancer PRS in the PGS catalog.

### Impact of individual level PRS uncertainty on relative risk and risk prediction

We applied multivariable logistic regression to discover the effect of PRS-based risk subgroups on lung cancer risk controlling for age, gender, and smoking history in the individuals who were identified with certainty. Stratified analyses by gender, smoking status, and lung cancer histology were similarly conducted. Lung cancer risk prediction models were constructed by integrating both PRS risk groups and other non-genetic baseline covariates, such as age, gender, and smoking history. The prediction model performance was evaluated using five-fold cross-validation based on the metric of AUC. To investigate the potential impact of individual-level uncertainty on risk prediction, we constructed a risk prediction model within the individuals identified with certainty (certainly high vs. low risk) and compared the model performance constructed in the high (*n* = 3006) and low (*n* = 3006) risk deciles identified by PRS-16-CV mean without taking individual level uncertainty into account under the exact same model specification.

## Results

Table 1 summarizes the baseline characteristics of 30,060 participants that were included in the study. Ever smokers were significantly enriched in lung cancer cases with a significantly longer median smoking pack-years (39 pack-years) compared to controls (13 pack-years). Among lung cancer patients, 76.4% of the cases were non-small cell lung cancer (NSCLC) with an enrichment of lung adenocarcinoma (38.1%). Three PRSs (PRS-16, PRS-16-CV, and PRS-Bayes) of lung cancer risk were calculated for each individual and statistically higher PRS mean (mPRS) were consistently observed in lung cancer patients compared to controls (mPRS-16 in lung cancer cases: 1.26, controls: 1.21, *P*-value < 2.2e−16; mPRS-16-CV in lung cancer cases: 1.21, controls: 1.16, *P*-value < 2.2e−16; mPRS-Bayes in lung cancer cases: − 0.04, controls: − 0.11, *P*-value < 2.2e−16), suggesting a potentially higher genetic risk in lung cancer patients (Additional file 2: Fig. S1).

**Table 1:** Baseline demographic information of the study population

Demographic information	Overall	Case	Control
	(*N* = 30,060)	(*n* = 17,166)	(*n* = 12,894)
**Gender**			
Female (*n*, %)	11,417 (38.0)	6785 (37.4)	4962 (38.5)
Male (*n*, %)	18,643 (62.0)	10,711 (62.4)	7932 (61.5)
**Age, mean (SD)**	62.9 (10.6)	63.7 (10.7)	62.0 (10.6)
**Histology**			
NSCLC (*n*, %)	13,116 (76.4)	13,116 (76.4)	-
ADC (*n*, %)	6539 (38.1)	6539 (38.1)	-
SCC (*n*, %)	4180 (24.4)	4180 (24.4)	-
Others (*n*, %)	2397 (14.0)	2397 (14.0)	-
SCLC (*n*, %)	1763 (10.3)	1763 (10.3)	-
Others (*n*, %)	2287 (13.3)	2287 (13.3)	-
**Smoking information**			
Never smoker (*n*, %)	5805 (19.3)	1702 (9.9)	4103 (31.8)
Former smoker (*n*, %)	11,497 (38.2)	6683 (38.9)	4814 (37.3)
Current smoker (*n*, %)	12,758 (42.4)	8781 (51.2)	3977 (30.8)
Pack-years, median (range)	29.4 (0.0, 315.0)	39.0 (0.0, 315.0)	13.0 (0.0, 260.0)

*SD* standard deviation; *NSCLC* non-small cell lung cancer; *ADC* adenocarcinoma, *SCC* squamous cell carcinoma; *SCLC* small cell lung cancer

### Characterization of lung cancer PRS uncertainty at the individual level

**Fig. 3:**
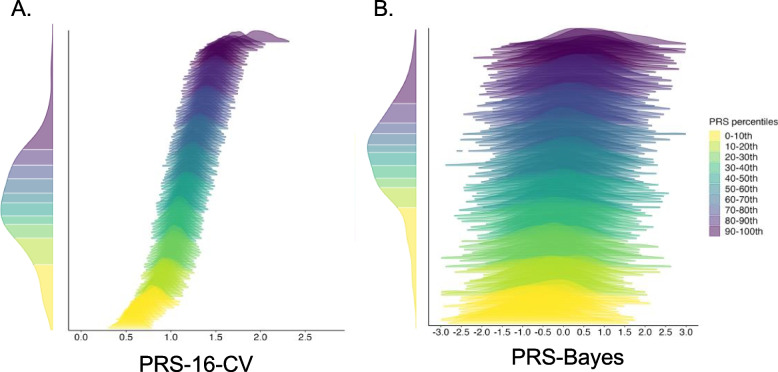
Individual-level distribution of PRS-16-CV and PRS-Bayes. **A** Individual-level PRS distributions obtained from PRS-16-CV. **B** Individual-level PRS distributions obtained from PRS-Bayes. For illustration purposes, here we only show the individual-level PRS distributions for 100 participants

The variability of lung cancer PRS at the individual level was evaluated by leveraging the CIs constructed in PRS-16-CV and PRS-Bayes. A considerable variation of PRS point estimates was observed for both methods. On average, a larger standard deviation (s.d.) of individual-level PRS distribution was observed in PRS-Bayes (mean s.d.: 0.88, 95% CI of s.d.: 0.68–1.11) compared to PRS-16-CV (mean s.d.: 0.12, 95% CI of s.d.: 0.09–0.15), suggesting a larger variability using PRS-Bayes at the individual level. For illustration purposes, we show the individual-level PRS distribution for both methods in 100 individuals (Fig. 3).

### Impact of Individual-level PRS uncertainty on lung cancer risk stratification

All individuals were stratified into PRS deciles and at a confidence/credible level of 95%. Using PRS-16-CV, only 25.0% (751/3006) of individuals with PRS point estimates in the lowest decile of PRS and 16.8% (505/3006) in the highest decile have their entire 95% CI fully contained in the lowest and highest decile, respectively, while PRS-Bayes was unable to find any eligible individual (Table 2). We further conducted sensitivity analysis by changing the population level threshold *t* and the confidence/credible level *p*. We varied the range of the confidence/credible level *p* from 0 to 100% and fixed the threshold at *t* = 10th or 5th percentile for the low-risk population and at *t* = 90th or 95th percentile for the high-risk population. The proportion of certainty was negatively correlated with the confidence/credible level *p* for both the high-risk and low-risk classification (Additional file 2: Fig. S2). A consistently lower percentage of certain classifications was observed in PRS-Bayes than in PRS-16-CV across all confidence/credible levels. Similar relationships between the proportion and the confidence/credible level *p* were observed across subgroups of gender, histology, and smoking status (Additional file 2: Fig. S3). The proportion of certainty decreases as more stringent threshold t and confidence/credible level *p* are specified.

**Table 2:** PRS-based rankings identified by PRS-16-CV and PRS-Bayes

PRS deciles	PRS-16-CV		PRS-Bayes	
	*n*	Mean rankings [range]	*n*	Mean rankings [range]
0th–10th	751	7th [0th, 66th]	-	34th [0th, 100th]
11th–20th	-	18th [0th, 76th]	-	41st [0th, 100th]
21st–30th	-	28th [1st, 85th]	-	44th [0th, 100th]
31st–40th	-	37th [2nd, 90th]	-	46th [0th, 100th]
41st–50th	-	46th [3rd, 94th]	-	49th [0th, 100th]
51st–60th	-	55th [5th, 97th]	-	51st [0th, 100th]
61st–70th	-	63th [7th, 98th]	-	54th [0th, 100th]
71st–80th	-	72nd [12nd, 99th]	-	56th [0th, 100th]
81st–90th	-	82nd [14th, 100th]	-	60th [0th, 100th]
91st–100th	505	92th [25th, 100th]	-	66th [0th, 100th]

The mean and range of the rankings for each individual within each decile identified by PRS-16-CV and PRS-Bayes were calculated. The column *n* indicates the number of individuals that can be identified with certainty. Using PRS-16-CV, 751 individuals in the lowest PRS decile and 505 individuals in the highest decile can be identified with certainty. In contrast, PRS-Bayes were not able to identify any individuals with certainty

Furthermore, the individual-level uncertainty greatly impacts PRS-based rankings. Each individual would have a distribution of the rankings for each PRS point estimate obtained from both PRS-16-CV and PRS-Bayes. We calculated the mean and range of the rankings for each individual. Substantial variability was also observed in the rankings identified by both methods for each PRS decile (Table 2). A wider range of rankings was observed for PRS-Bayes compared to using PRS-16-CV. The minimal range of the rankings was observed in the lowest decile (mean ranking 7th, range: 0–66th) identified by PRS-16-CV, and individuals above the 90th percentile of PRS can be anywhere from the 25th–100th percentile. In contrast, individuals in each PRS decile can be anywhere from the 0th–100th using PRS-Bayes when their CI is taken into consideration.

To answer the question of whether individuals would be commonly assigned into the same genetic risk categories, we counted the number of overlapped individuals that were commonly identified using different PRS estimators of PRS-16, PRS-16-CV, and PRS-Bayes (Table 3). Overall, the degree of overlap decreases as the population level threshold increases. PRS-16 and PRS-16-CV identified a fair number of overlapped individuals as the same SNP loci were utilized. Two thousand four hundred seventy (82%) individuals were concordantly classified as high risk (> 90th percentile) using PRS-16 and PRS-16-CV, while PRS-Bayes agreed with either PRS-16-CV or PRS-16 on 19% of high-risk (> 90th percentile) stratification. In addition, we assessed the concordance of 22 lung cancer PGSs available in the PGS catalog, and similar modest correlations in PRS-based risk stratification were observed using PGS estimate (Additional file 2: Fig. S4-7). Taken together, substantial disagreement was observed for lung cancer PRS at the individual level using different PRS estimators.

**Table 3:** Overlap of commonly identified individuals using different PRS estimators

Thresholds	PRS-16 vs. PRS-16-CV (*n*,%)	PRS-16 vs. PRS-Bayes (*n*,%)	PRS-16-CV vs. PRS-Bayes (*n*,%)
> 60th	10,865 (90)	5998 (50)	6068 (50)
> 70th	7942 (88)	3690 (41)	3726 (41)
> 80th	5161 (85)	1892 (31)	1905 (32)
> 90th	2470 (82)	583 (19)	573 (19)
> 95th	1218 (78)	174 (12)	163 (11)

The number and percentage of individuals that can be commonly identified with the same PRS-based risk groups by any of the two PRS estimators (PRS-16 vs. PRS-16-CV; PRS-16 vs. PRS-Bayes; PRS-16-CV vs. PRS-Bayes) were shown as the threshold of PRS risk increases

### Impact of individual-level uncertainty on relative risk of PRS deciles on lung cancer

Next, we evaluated its impact on lung cancer risk in individuals identified by PRS-16-CV taking individual-level uncertainty into account. In contrast to risk stratification based on PRS mean, only individuals in the top and lowest deciles were able to be identified with certainty when taking variance in PRS estimates into account; hence, the relative risk was only evaluated in the two PRS-based risk subgroups. An increased effect size for PRS CI (OR = 2.73, 95% CI: 2.12–3.50, *P*-value = 4.13 × 10^−15^) was compared to using PRS mean (OR = 2.23, 95% CI: 1.99–2.49, *P*-value = 5.70 × 10^−46^) (Table 4). Similar improvement was observed in stratified analyses by gender, lung cancer histology, and smoking status (Table 5). The largest increase in relative risk of lung cancer from OR = 2.63 to OR = 4.22 was observed in never smokers, suggesting a potentially larger genetic contribution to lung cancer and thus more susceptible to individual-level PRS uncertainty.

**Table 4:** Impact of individual-level uncertainty on relative risk of PRS deciles on lung cancer risk

PRS deciles	PRS CI-based risk stratification			PRS mean-based risk stratification		
	OR (95% CI)	*P*-value	*n*	OR (95% CI)	*P*-value	*n*
0th–10th	1 (reference group)	-	751	1 (reference group)		3006
11th–20th	-	-		1.15 (1.04, 1.29)	8.80e−03	3006
21st–30th	-	-		1.31 (1.17, 1.45)	9.70e−07	3006
31st–40th	-	-		1.31 (1.18, 1.46)	6.70e−07	3006
41st–50th	-	-		1.44(1.30, 1.61)	2.40e−11	3006
51st–60th	-	-		1.54 (1.39, 1.72)	3.00e−15	3006
61st–70th	-	-		1.55 (1.39, 1.72)	2.00e−15	3006
71st–80th	-	-		1.80 (1.61, 2.01)	3.60e−26	3006
81st–90th	-	-		1.76 (1.58, 1.96)	2.40e−24	3006
91st–100th	2.73 (2.12, 3.50)	4.13e−15	505	2.23 (1.99, 2.49)	5.70e−46	3006

Odds ratio of lung cancer comparing different PRS deciles identified by PRS CI-based approach taking individual level uncertainty into account and by PRS mean were shown. As the PRS-16-CV CI-based approach was only able to identify individuals in the lowest (*n* = 751) and highest decile (*n* = 505) with certainty, the analysis was only conducted in the two subsets. In contrast, using the PRS-16-CV mean, the analyses were conducted in each PRS decile compared to the lowest one. The detailed sample sizes that were included in each analysis were noted in column *n*. Odds ratio (OR) and 95% confidence intervals (CI) are shown. All models were adjusted for age, gender, and smoking status

**Table 5:** Impact of individual-level uncertainty on the relative risk of PRS deciles on lung cancer risk in stratified analyses by gender, lung cancer histology, and smoking status

	Subgroups	90th vs. 10th (PRS CI-based)			90th vs. 10th (PRS mean-based)		
		OR (95% CI)	*P*-value	*n*	OR (95% CI)	*P*-value	*n*
**Gender**	**Male**	2.63 (1.91, 3.62)	3.09e−09	772	2.06 (1.79, 2.37)	2.80e−24	3731
	**Female**	2.88 (1.92, 4.32)	2.83e−07	484	2.54 (2.12, 3.04)	3.39e−24	2281
**Histology**	**NSCLC**	2.84 (2.19, 3.70)	6.14e−15	1117	2.31 (2.06, 2.60)	3.53e−45	5199
	**ADC**	3.70 (2.69, 5.09)	8.71e−16	842	2.76 (2.39, 3.18)	9.60e−45	3874
	**SCC**	2.11 (1.45, 3.07)	8.93e−05	752	1.71 (1.45, 2.02)	2.61e−10	3406
	**SCLC**	2.53 (1.43, 4.46)	1.38e−03	630	1.86 (1.47, 2.36)	2.01e−07	2906
**Smoking**	**Never**	4.22 (2.25, 7.92)	7.25e−06	223	2.63 (2.02, 3.43)	9.94e−13	1125
	**Former**	2.25 (1.54, 3.28)	2.43e−05	494	2.18 (1.83, 2.59)	1.19e−18	2280
	**Current**	2.74 (1.84, 4.10)	8.28e−07	539	2.13 (1.79, 2.52)	3.88e−18	2607

For the PRS CI-based approach, the stratified analyses were only conducted in the individuals that can be identified with certainty. The detailed sample sizes that were included in each analysis were noted in column *n*. Odds ratio (OR) and 95% confidence intervals (CI) are shown. *NSCLC*, non-small cell lung cancer; *ADC*, adenocarcinoma, *SCC*, squamous cell carcinoma; *SCLC*, small cell lung cancer

### Impact of individual-level PRS uncertainty on lung cancer risk prediction

Finally, we evaluated the impact of the individual-level PRS uncertainty on lung cancer risk prediction by including the two PRS-based risk subgroups identified by PRS-16-CV. Prediction models were constructed based on PRS-based risk subgroups and non-genetic risk factors of age, gender, and smoking history. An improved prediction model performance was consistently observed in all models when considering individual-level uncertainty compared to using PRS mean solely (Table 6). The discriminative performance improved as we gradually incorporated non-genetic predictors into the model in a sequence of age, gender, and smoking information. The highest AUC (0.73, 95% CI = 0.72–0.74) was achieved by the model whose predictors were a combination of PRS-based risk categories and non-genetic predictors of age, gender, and detailed smoking pack years. Taking individual-level uncertainty of genetic risk into account in conjunction with non-genetic risk factors improves lung cancer risk prediction.

**Table 6:** Impact of individual-level uncertainty on lung cancer risk prediction performance

Prediction models	PRS CI-based (*n* = 1256)	PRS mean-based (*n* = 6012)
	AUC (95% CI)	AUC (95% CI)
**PRS > 90th vs. PRS < 10th (PRS risk)**	0.6076 (0.6055, 0.6082)	0.5925 (0.5903, 0.5948)
**PRS risk + age**	0.6363 (0.63196, 0.63688)	0.6094 (0.6093, 0.6097)
**PRS risk + age + gender**	0.6347 (0.6198, 0.6407)	0.6088 (0.6041, 0.6106)
**PRS risk + age + gender + smoking status**	0.7104 (0.6980, 0.7191)	0.6884 (0.6852, 0.6905)
**PRS risk + age + gender + packyears**	0.7299 (0.7179, 0.7388)	0.7182 (0.7154, 0.7202)
**Age + gender + smoking status**	0.6584 (0.6562, 0.6610)	0.6602 (0.6558, 0.6621)

The prediction model performances incorporating different risk factors of PRS-based risk subgroup, age, gender, and smoking history were evaluated in subsets of individuals that were identified by the PRS CI-based approach and by the PRS mean-based approach. For the PRS CI-based approach, the models were constructed and evaluated in the individuals that can be identified with certainty (*n* = 1256). As a comparison, we constructed the same models and evaluated in the individuals that were classified as the lowest and highest risk by PRS-16-CV mean (*n* = 6012). The model performance was evaluated using five-fold cross-validation. Area under the curve (AUC) and 95% confidence intervals (CI) are shown

## Discussion

Our work explored lung cancer PRS uncertainty for individuals in populations with European ancestry using both GWAS-derived SNPs and LDpred2 and demonstrated that individual-level PRS uncertainty greatly impacts PRS-based rankings, risk stratification, and ultimately prediction of lung cancer risk. The substantial recent interest in translating lung cancer PRS and other predictive tools like deep learning models from low radiation dose chest computed tomography (LDCT) for future lung cancer risk prediction necessitates a careful assessment of individual-level uncertainty to truly accomplish personalized risk assessment [35, 36]. Taking individual-level uncertainty into PRS-based risk stratification and prediction provides more accurate risk stratification and improves the ability to identify high-risk subjects and recommend LDCT screening with more certainty. It is imperative to develop more stable PRS and account for uncertainty at the individual level in risk stratification to help ensure reliable clinical applications of PRS in real-world settings.

A critical concern of PRS application is delivering inaccurate risk estimates at the individual level, and wrongly categorizing an individual as low or high genetic risk based on unstable PRS estimates. The downstream effect could be inappropriate or even contradictory medical advice or clinical decisions [25]. Our study shows substantial disagreement in risk categorization using different lung cancer PRSs (PRS-16, PRS-16-CV, and PRS-Bayes), suggesting that individuals that were identified as very high risk by one PRS method may not be classified as such by another. Similar modest correlations were observed for another 22 lung cancer PRS in the PGS catalog. This issue is not unique to lung cancer, as large discordances have also been found in breast cancer, hypertension, and dementia using approaches to calculate PRS in a white British population [22]. More complex traits or diseases and different ancestries between the discovery and target population of GWAS may lead to even more profound inconsistency at the individual level [24]. PRS needs to be reliable and reproducible if it is going to inform personalized decision-making in clinical settings.

Comparing the two PRS generative methods, a much larger variability was observed in PRS-Bayes, resulting in no individuals can be identified with sufficient certainty. Ding et al. also found only a limited proportion of individuals are classified as high risk with certainty across 13 traits in UK Biobank [21]. PRS-Bayes includes a much greater number of non-zero weight SNPs (~2000 SNPs) with small effect sizes and this may explain a higher proportion of SNP-based heritability compared to PRS-16-CV (16 SNPs). In the meanwhile, the numerous SNPs with small effect sizes may contribute to a higher variance in PRS estimates. PRS-Bayes may perform better in terms of lung cancer risk prediction at the population level as the accuracy is determined by the proportion of phenotypic variance explained by variants included and the improved population prediction error. On the other hand, PRS-16-CV is relatively parsimonious and contains only those that have been robustly validated SNPs and have a minor individual-level variance. Additionally, we constructed and evaluated the individual-level PRS uncertainty and population-level prediction accuracy using nine experimentally validated SNPs that are associated with lung cancer risk [37] (Additional file 1: Table S4). More individuals can be identified with certainty while similar prediction accuracy was achieved (Additional file 1: Table S5-6). This may suggest that experimentally validated fine-mapped variants may be more likely to be biologically causal variants and thus have stable effects and PRS risk stratification performance at the individual level for lung cancer prediction given the similar population-level prediction accuracy. A trade-off between these methods may be manifested by the compromise between prediction performance at the population level and stability at the individual level, and this needs to be considered cautiously when developing new PRS methods tailoring for different purposes and applications [21].

Given the individual-level PRS uncertainty, it is imperative to cautiously interpret and implement it in clinical applications. From our analyses, using the PRS-based risk subgroup alone resulted in the lowest AUC, and including other non-genetic factors of age, gender, and smoking history largely improved the lung cancer risk prediction performance. PRS stand-alone is imperfect and the use of PRS as a covariate or effect modifier in conjunction with non-genetic risk factors may inform better outcomes [22, 38]. Despite modest improvement in prediction accuracy using PRS-based risk subgroups taking individual-level uncertainty into account, likely due to the limited sample sizes of eligible individuals, it attains both PRS stability and predictive accuracy given the data. Also, it reflects the situation that it is challenging for the current PRS approaches to balance reproducible PRS for individuals with sufficient certainty and high prediction accuracy at the population level. It is crucial and pressing to develop guidelines in constructing PRS to minimize the extent to which individuals could be provided with imprecise or contradictory clinical advice and intervention as well as PRS reporting standards in light of the patient and primary care providers’ perspective [25].

There are several improvements and future directions for the present study. First, more statistical approaches to construct lung cancer PRS, e.g., regularization-based methods, can be included [39]. Second, we did not assess the potential interactions between lung cancer PRS and non-genetic risk factors when taking individual-level uncertainty into account, and we would expect more uncertainty arising from environmental factors as well as from the gene-environment interactions in predicting the risk of lung cancer [40]. Further research is needed to assess the impact on performance when using commonly applied LD clumping methods for SNP selection, particularly with varying parameters. Lastly, our study was conducted in a population of European ancestry; thus, the generalizability of the results in other populations of different genetic ancestries is a concern given the discrepancy of genetic structures for causal variants, allele frequencies, and LD patterns across ancestries. To realize the equitable and reliable potential of PRS, it is necessary to carry out larger, multi-ancestry GWAS and further investigate the uncertainty at the individual level in PRS.

## Conclusions

Our study characterized the uncertainty of lung cancer PRS at the individual level and evaluated its impact on subsequent PRS-based ranking, risk stratification, and prediction in populations with European ancestry. It is imperative to develop reproducible PRS, reliable PRS-based clinical applications as well as guidelines to report and communicate PRS to patients and their primary physicians.

### Supplementary Information

**Additional file 1: Table S1** Participating samples and their origin sites in the ILCCO lung cancer OncoArray project. **Table S2** Information of 16 GWAS-derived lung cancer SNPs included in PRS-16 and PRS-16-CV. **Table S3** Information of 19 GWAS-derived lung cancer SNPs that have been validated in Caucasians from prior studies. **Table S4** Fine-mapped lung cancer risk variants that have been experimentally validated. **Table S5** Rankings of individuals identified by three PRS models. **Table S6** AUC of predicting lung cancer in the confident individuals.

**Additional file 2: Figure S1** The population-level distribution of lung cancer risk PRSs. **Figure S2** PRS CI-based stratification uncertainty. Figure S3. PRS-based stratification uncertainty in subgroups. **Figure S4-S7** Proportions of concordant individuals by 22 lung cancer PRSs in the PGS catalog based on different population thresholds.

## Data Availability

Genotype and phenotype data used in this work are available from dbGaP (https://www.ncbi.nlm.nih.gov/projects/gap/cgi-bin/study.cgi?study_id=phs001273.v4.p2; phs001273.v3.p2; )[2].
