# Supplementary material for: Impact of individual level uncertainty of lung cancer polygenic risk score (PRS) on risk stratification
Source: Genome Med. 2024 Feb 5;16:22. doi: 10.1186/s13073-024-01298-4 (PMC10840262; doi:10.1186/s13073-024-01298-4)
Supplement: Supplementary file 1 — Additional file 1: Table S1 Participating samples and their origin sites in the ILCCO lung cancer OncoArray project. Table S2 Information of 16 GWAS-derived lung cancer SNPs included in PRS-16 and PRS-16-CV. Table S3 Information of 19 GWAS-derived lung cancer SNPs that have been validated in Caucasians from prior studies. Table S4 Fine-mapped lung cancer risk variants that have been experimentally validated. Table S5 Rankings of individuals identified by three PRS models. Table S6 AUC of predicting lung cancer in the confident individuals. [file 13073_2024_1298_MOESM1_ESM.docx]

**Table S1:** Participating samples and their origin sites in the ILCCO lung cancer OncoArray project

| **Sites** | **Total (N=30060)** | **Cases (n=17166)** | **Controls (n=12894)** |
| --- | --- | --- | --- |
| ATBC | 1831 | 1115 | 716 |
| CANADA | 716 | 261 | 455 |
| CAPUA | 1425 | 729 | 696 |
| EAGLE | 3575 | 1800 | 1775 |
| CARET | 1069 | 546 | 523 |
| LLP | 924 | 441 | 483 |
| GERMANY | 1572 | 988 | 584 |
| HLCS | 3544 | 2892 | 652 |
| NICCC-LCA | 1211 | 680 | 531 |
| KENTUCKY | 230 | 97 | 133 |
| MDACC | 2011 | 1013 | 998 |
| MEC | 461 | 226 | 235 |
| NELCS | 338 | 164 | 174 |
| NIJMEGEN | 853 | 399 | 454 |
| NORWAY | 746 | 323 | 423 |
| PLCO | 2272 | 1354 | 918 |
| RESOLUCENT | 958 | 576 | 382 |
| L2 | 2081 | 1014 | 1067 |
| TAMPA | 255 | 104 | 151 |
| TLC | 427 | 427 | 0 |
| MSH-PMH | 2389 | 1408 | 981 |
| BioVU | 1172 | 609 | 563 |

ATBC, The Alpha-Tocopherol, Beta-Carotene Cancer Prevention; CANADA, Canadian screening study; CAPUA, Cancer de Pulmon en Asturias; EAGLE, Environment and Genetics in Lung Cancer Study Etiology; CARET, The Carotene and Retinol Efficacy; LLP, Liverpool Lung Cancer Project; GERMANY, German lung cancer study; HLCS, Harvard Lung Cancer Study; NICCC-LCA, Clalit National Israeli Cancer Control Center- lung cancer study; KENTUCKY, Kentucky Lung Cancer Research Initiative; MDACC, MD Anderson Cancer Center Study; MEC, Multiethnic Cohort Study; NELCS, New England Lung Cancer Study; NIJMEGEN, The Nijmegen Lung Cancer Study; Norway, Norway Lung Cancer Study; PLCO, The Prostate, Lung, Colorectal and Ovarian Cancer Screening Trial; RESOLUCENT, The Resource for the Study of Lung Cancer Epidemiology in North Trent; L2, The IARC L2 Study; TAMPA, Tampa Lung Cancer Study; TLC, Total Lung Cancer: Molecular Epidemiology of Lung Cancer Survival; MSH-PMH, Mount Sinai Hospital-Princess Margaret Hospital Study; BioVU, The Vanderbilt Lung Cancer Study.

**Table S2:** Detailed information of 16 GWAS-derived lung cancer SNPs included in PRS-16 and PRS-16-CV.

| **SNP** | **Chr** | **Position** | **Locus** | **Gene** | **Reference allele** | **Effect allele** | **EAF** | **Beta** |
| --- | --- | --- | --- | --- | --- | --- | --- | --- |
| rs71658797 | 1 | 77501822 | 1p31.1 | *AK5* | T | A | 0.091 | 0.128 |
| rs13080835 | 3 | 189639410 | 3q28 | *TP63* | T | G | 0.481 | 0.057 |
| rs7705526 | 5 | 1285859 | 5p15.33 | *TERT* | C | A | 0.36 | 0.117 |
| rs2853677 | 5 | 1287079 | 5p15.33 | *TERT* | A | G | 0.447 | 0.111 |
| rs465498 | 5 | 1325688 | 5p15.33 | *CLPTM1L* | G | A | 0.401 | 0.141 |
| rs3115672 | 6 | 31760120 | 6p21.33 | *MSH5* | C | T | 0.088 | 0.166 |
| rs6920364 | 6 | 166962978 | 6q27 | *MIR3939* | G | C | 0.487 | 0.068 |
| rs11780471 | 8 | 27487202 | 8p21.2 | *CHRNA2* | A | G | 0.052 | 0.141 |
| rs4236709 | 8 | 32552592 | 8p12 | *NRG1* | A | G | 0.216 | 0.064 |
| rs885518 | 9 | 21830158 | 9p21.3 | *MTAP* | A | G | 0.134 | 0.088 |
| rs62560775 | 9 | 22052069 | 9p21.3 | *CDKN2B-AS1* | A | G | 0.096 | 0.100 |
| rs1056562 | 11 | 118254910 | 11q23.3 | *MPZL2* | C | T | 0.476 | 0.066 |
| rs7953330 | 12 | 889653 | 12p13.33 | *WNK1* | C | G | 0.295 | 0.087 |
| rs11571833 | 13 | 32398489 | 13q13.1 | *BRCA2* | A | T | 0.01 | 0.472 |
| rs66759488 | 15 | 47285254 | 15q21.1 | *SEMA6D* | G | A | 0.354 | 0.068 |
| rs77468143 | 15 | 49084427 | 15q21.1 | *SECISBP2L* | G | T | 0.236 | 0.083 |

**Table S3:** Detailed information of 19 GWAS-derived lung cancer SNPs that have been validated in Caucasians from prior studies.

| **SNP** | **Chr** | **Position** | **Locus** | **Gene** | **Reference allele** | **Effect allele** | **EAF** | **Beta** |
| --- | --- | --- | --- | --- | --- | --- | --- | --- |
| rs71658797 | 1 | 77501822 | 1p31.1 | *AK5* | T | A | 0.091 | 0.128 |
| rs13080835 | 3 | 189639410 | 3q28 | *TP63* | T | G | 0.481 | 0.057 |
| rs7705526 | 5 | 1285859 | 5p15.33 | *TERT* | C | A | 0.36 | 0.117 |
| rs2853677 | 5 | 1287079 | 5p15.33 | *TERT* | A | G | 0.447 | 0.111 |
| rs465498 | 5 | 1325688 | 5p15.33 | *CLPTM1L* | G | A | 0.401 | 0.141 |
| rs3115672 | 6 | 31760120 | 6p21.33 | *MSH5* | C | T | 0.088 | 0.166 |
| rs6920364 | 6 | 166962978 | 6q27 | *MIR3939* | G | C | 0.487 | 0.068 |
| rs11780471 | 8 | 27487202 | 8p21.2 | *CHRNA2* | A | G | 0.052 | 0.141 |
| rs4236709 | 8 | 32552592 | 8p12 | *NRG1* | A | G | 0.216 | 0.064 |
| rs885518 | 9 | 21830158 | 9p21.3 | *MTAP* | A | G | 0.134 | 0.088 |
| rs62560775 | 9 | 22052069 | 9p21.3 | *CDKN2B-AS1* | A | G | 0.096 | 0.100 |
| rs1056562 | 11 | 118254910 | 11q23.3 | *MPZL2* | C | T | 0.476 | 0.066 |
| rs7953330 | 12 | 889653 | 12p13.33 | *WNK1* | C | G | 0.295 | 0.087 |
| rs11571833 | 13 | 32398489 | 13q13.1 | *BRCA2* | A | T | 0.01 | 0.472 |
| rs66759488 | 15 | 47285254 | 15q21.1 | *SEMA6D* | G | A | 0.354 | 0.068 |
| rs77468143 | 15 | 49084427 | 15q21.1 | *SECISBP2L* | G | T | 0.236 | 0.083 |
| rs55781567 | 15 | 78565644 | 15q25.1 | *CHRNA5* | C | G | 0.349 | 0.260 |
| rs8042374 | 15 | 78615690 | 15q25.1 | *CHRNA3* | G | A | 0.267 | 0.232 |
| rs56113850 | 19 | 40847202 | 19q13.2 | *CYP2A6* | T | C | 0.437 | 0.123 |

**Table S4:** Fine-mapped lung cancer risk variants that have been experimentally validated.

| **rsID** | **Chr** | **BP** | **Locus** | **Mechanisms** | **PubMed ID** |
| --- | --- | --- | --- | --- | --- |
| rs13314271 | 3 | 189357602 | 3q28 | DNA damage repair and cellular stress response | 24880342 |
| rs401681 | 5 | 1322087 | 5p15.33 | Telomere length and chromosomal integrity | 18978787 |
| rs4975616 | 5 | 1315660 | 5p15.33 | Telomere length and chromosomal integrity | 19654303 |
| rs3117582 | 6 | 31620520 | 6p21.33 | Major histocompatibility complex region | 19654303,  18978787, |
| rs11571833 | 13 | 32972626 | 13q13.1 | DNA damage repair and cellular stress response | 24880342 |
| rs8034191 | 15 | 78806023 | 15q25.1 | Smoking behavior and nicotine metabolism | 19654303,  18385738, 18385676, 18780872 |
| rs1051730 | 15 | 78894339 | 15q25.1 | Smoking behavior and nicotine metabolism | 30104567,  18978790 |
| rs8042374 | 15 | 78908032 | 15q25.1 | Smoking behavior and nicotine metabolism | 18978787 |
| rs17879961 | 22 | 29121087 | 22q12.1 | DNA damage repair and cellular stress response | 24880342 |

**Table S5: Rankings of individuals identified by three PRS models.**

| **PRS deciles** | **PRS-16-CV** | | **PRS-Bayes** | | **PRS-FM-CV** | |
| --- | --- | --- | --- | --- | --- | --- |
|  | **n** | **mean rankings [range]** | **n** | **mean rankings [range]** | **n** | **mean rankings [range]** |
| 0-10^th^ | 757 | 7^th^ [0^th^, 66^th^] | - | 34^th^ [0^th^, 100^th^] | 1740 | 5^th^, [0^th^, 68^th^] |
| 10^th^-20^th^ | - | 18^th^ [0^th^, 76^th^] | - | 41^st^ [0^th^, 100^th^] | 211 | 15^th^, [0^th^, 61^st^] |
| 20^th^-30^th^ | - | 28^th^ [1^st^, 85^th^] | - | 44^th^ [0^th^, 100^th^] | 30 | 25^th^, [2^nd^, 71^st^] |
| 30^th^-40^th^ | - | 37^th^ [2^nd^, 90^th^] | - | 46^th^ [0^th^, 100^th^] | 389 | 35^th^, [4^th^, 82^nd^] |
| 40^th^-50^th^ | - | 46^th^ [3^rd^, 94^th^] | - | 49^th^ [0^th^, 100^th^] | 350 | 45^th^, [7^th^, 86^th^] |
| 50^th^-60^th^ | - | 55^th^ [5^th^, 97^th^] | - | 51^st^ [0^th^, 100^th^] | 304 | 55^th^, [10^th^, 91^st^] |
| 60^th^-70^th^ | - | 63^rd^ [7^th^, 98^th^] | - | 54^th^ [0^th^, 100^th^] | 44 | 65^th^, [17^th^, 94^th^] |
| 70^th^-80^th^ | - | 72^nd^ [12^nd^, 99^th^] | - | 56^th^ [0^th^, 100^th^] | 79 | 75^th^, [27^th^, 97^th^] |
| 80^th^-90^th^ | - | 82^nd^ [14^th^, 100^th^] | - | 60^th^ [0^th^, 100^th^] | 502 | 85^th^, [47^th^, 100^th^] |
| 90^th^-100^th^ | 505 | 92^nd^ [25^th^, 100^th^] | - | 66^th^ [0^th^, 100^th^] | 1776 | 95^th^, [65^th^, 100^th^] |

*** n indicates the number of individuals that can be identified with certainty by different PRS approaches.

**Table S6: AUC of predicting lung cancer in the confident individuals.**

|  | **PRS-FM-CV (n=1262)** | **PRS-16-CV (n=1262)** |
| --- | --- | --- |
| **90^th^ vs. 10^th^ percentile** | **AUC 95% CI** | **AUC 95% CI** |
|  | 0.6986 (0.6594, 0.7404) | 0.6994 (0.6568,0.7439) |

***Since individuals with certainty can only be identified in the lowest and the highest percentile by PRS-16-CV, we compared the AUC of predicting lung cancer along with age, sex, and smoking status in these two groups after down-sampling in PRS-FM-CV to make sure the same sample size was utilized.
