# Supplementary material for: Impact of individual level uncertainty of lung cancer polygenic risk score (PRS) on risk stratification
Source: Genome Med. 2024 Feb 5;16:22. doi: 10.1186/s13073-024-01298-4 (PMC10840262; doi:10.1186/s13073-024-01298-4)
Supplement: Supplementary file 2 — Additional file 2: Figure S1 The population-level distribution of lung cancer risk PRSs. Figure S2 PRS CI-based stratification uncertainty. Figure S3. PRS-based stratification uncertainty in subgroups. Figure S4-S7 Proportions of concordant individuals by 22 lung cancer PRSs in the PGS catalog based on different population thresholds. [file 13073_2024_1298_MOESM2_ESM.docx]

**Figure S1** The population-level distribution of lung cancer risk PRSs by lung cancer cases and controls. A. PRS-16; B. PRS-16-CV; C. PRS-Bayes.

**Figure S2** PRS CI-based stratification uncertainty using PRS-Bayes and PRS-16-CV at different thresholds and confidence/credible levels. A. Proportion of certain risk stratification for high-risk individuals; Confidence/credible levels vary from 0 to 100% at the x axis and two thresholds are selected as *t*=90^th^ and *t*=95^th^ percentiles. B. Proportion of certain risk stratification for low-risk individuals. Thresholds for low-risk stratification are fixed at *t*=10^th^ and *t*=5^th^ percentile.

**Figure S3** PRS-based stratification uncertainty in subgroups at t=90^th^ percentile. A-B) The proportion of PRS-based stratification that was certain for gender subgroups; C-D) the proportion of certain stratification for histological subgroups, including non-small cell lung cancer (NSCLC) and small cell lung cancer (SCLC); E-F) The proportion of certain stratification for smoking status, including never, former, and current smokers.


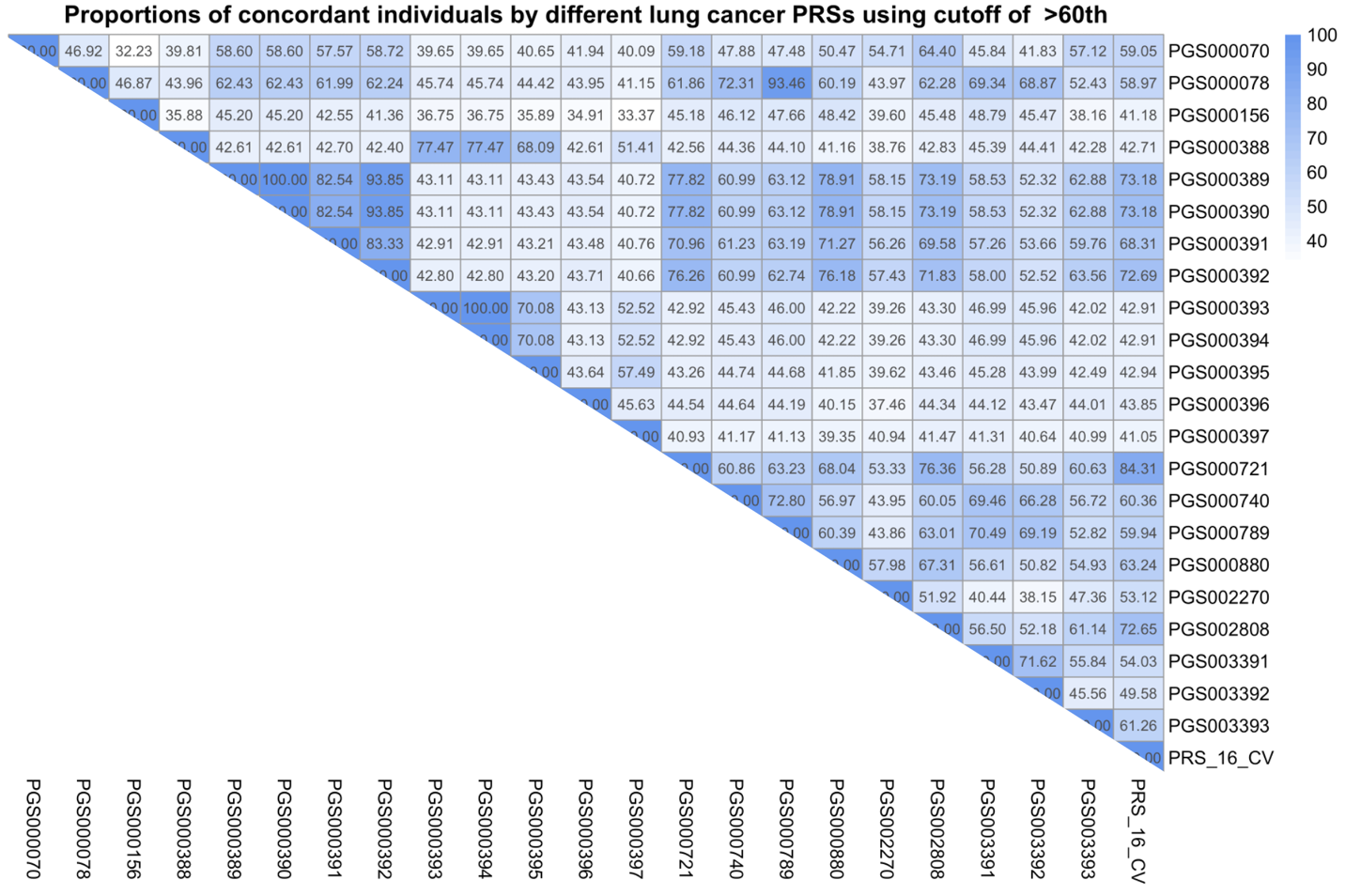


**Figure S4 Proportions of concordant individuals by 22 lung cancer PRSs in the PGS catalog based on population threshold of >60^th^ percentile**

Concordance of PRS-based risk stratification identified by 22 different lung cancer PRS PGS catalog was assessed using population level threshold of > 60^th^ percentile.


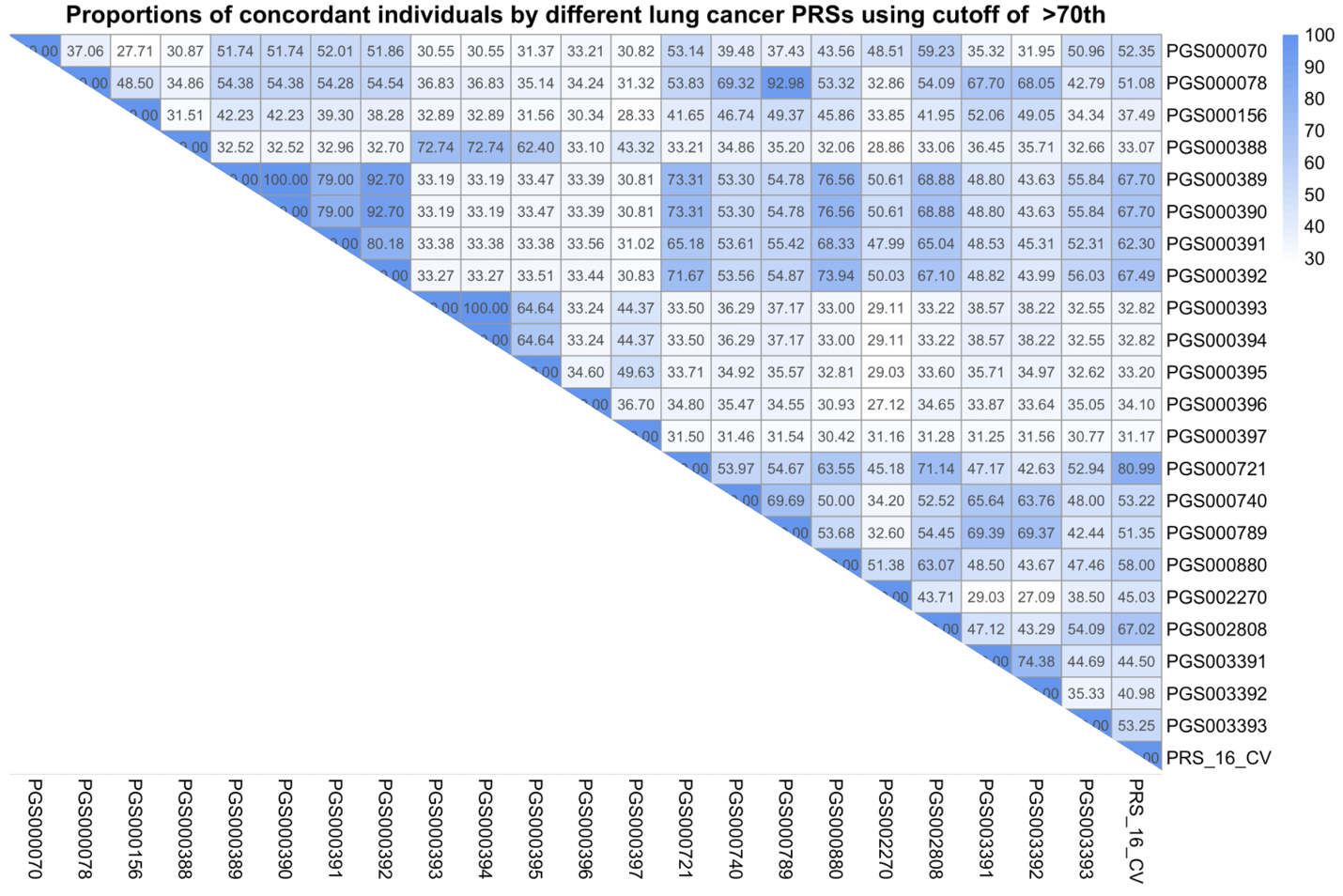


**Figure S5 Proportions of concordant individuals by 22 lung cancer PRSs in the PGS catalog based on population threshold of >70^th^ percentile**

Concordance of PRS-based risk stratification identified by 22 different lung cancer PRS PGS catalog was assessed using population level threshold of > 70^th^ percentile.


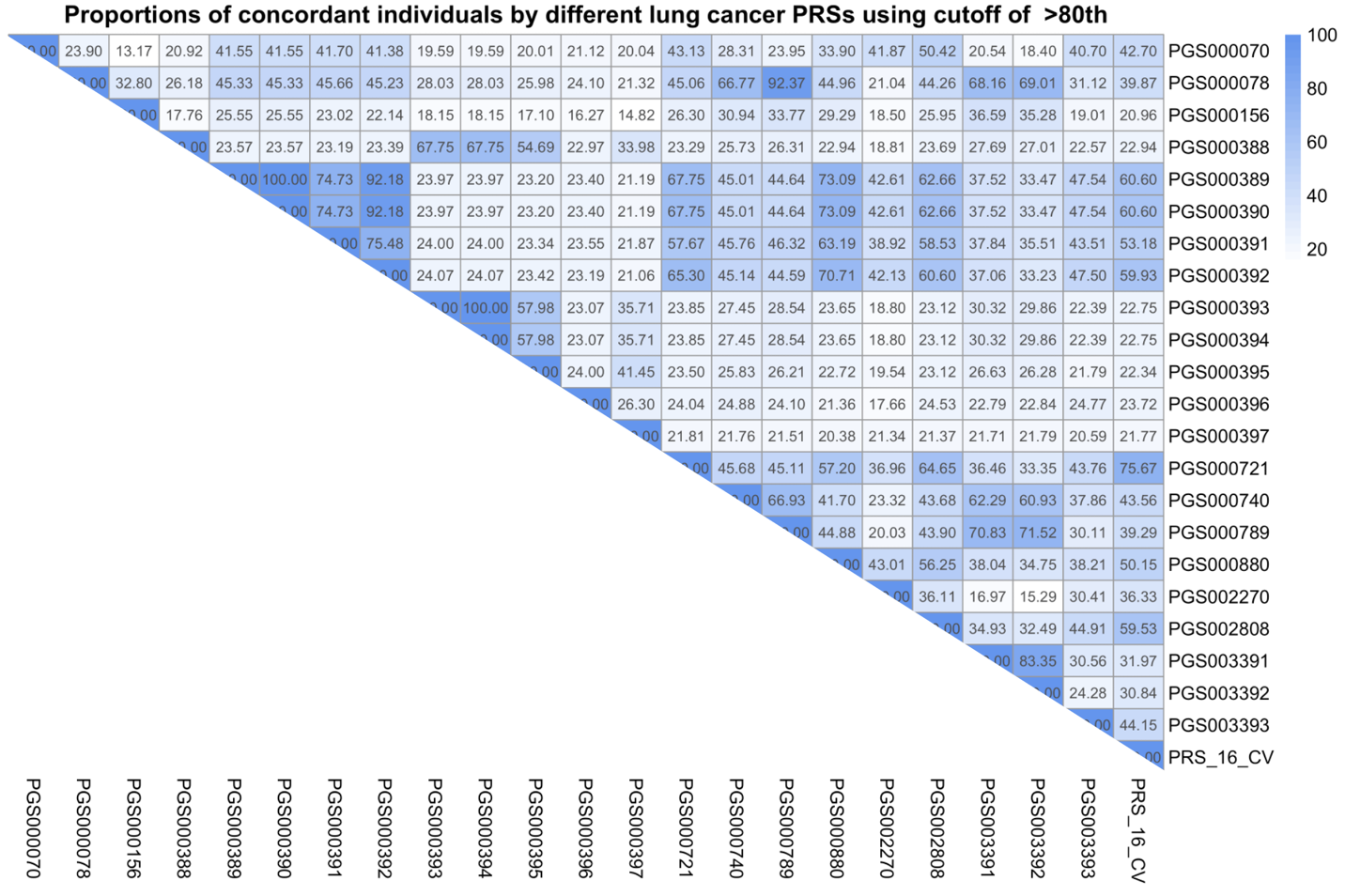


**Figure S6 Proportions of concordant individuals by 22 lung cancer PRSs in the PGS catalog based on population threshold of >80^th^ percentile**

Concordance of PRS-based risk stratification identified by 22 different lung cancer PRS PGS catalog was assessed using population level threshold of > 80^th^ percentile.


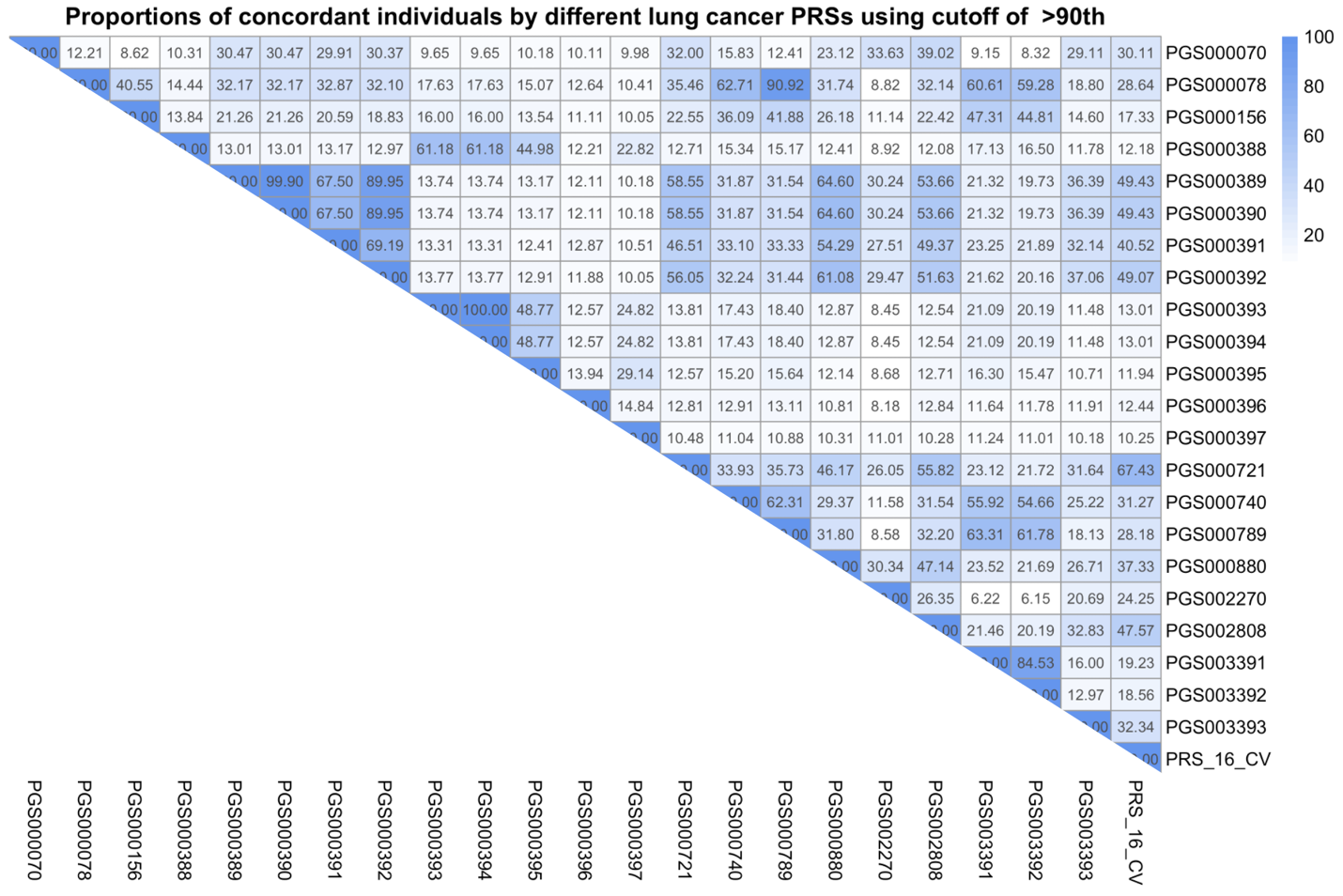


**Figure S7 Proportions of concordant individuals by 22 lung cancer PRSs in the PGS catalog based on population threshold of >90^th^ percentile**

Concordance of PRS-based risk stratification identified by 22 different lung cancer PRS PGS catalog was assessed using population level threshold of > 90^th^ percentile.
